# Magical thinking and moral injury: exclusion culture in psychiatry

**DOI:** 10.1192/bjb.2021.86

**Published:** 2022-02

**Authors:** Chloe Beale

**Affiliations:** 1 East London NHS Foundation Trust, London, UK; 2 Homerton University Hospital NHS Foundation Trust, London, UK

**Keywords:** Education and training, ethics, risk assessment, stigma and discrimination, suicide

## Abstract

This is an article about exclusion. We might not like to admit it – even fail to realise it – but National Health Service (NHS) mental health service structures have become increasingly focused on how to deny people care instead of help them to access it. Clinicians learn the art of self-delusion, convincing ourselves we are not letting patients down but, instead, doing the clinically appropriate thing. Well-meant initiatives become misappropriated to justify neglect. Are we trying to protect ourselves against the knowledge that we're failing our patients, or is collusion simply the easiest option? Problematic language endemic in psychiatry reveals a deeper issue: a culture of fear and falsehood, leading to iatrogenic harm. An excessively risk-averse and under-resourced system may drain its clinicians of compassion, losing sight of the human being behind each ‘protected’ bed and rejected referral.

## The language of exclusion

Choice of words in clinical work and documentation can betray troubling attitudes, personal values and fears. Given that psychoanalytic theory remains a core component of psychiatric training, we could do better at recognising our own defences. We have developed an entire lexicon of weasel words and magical thinking that we pass between generations and disciplines. It would be hard to come up with an exhaustive list of lies we tell ourselves in psychiatric practice; this does not claim to be one. Neither is this the first time anyone has pointed out the problematic language used in psychiatry.^1^ Patients are, of course, acutely aware of the absurdity.^2^,^3^ There are many which should be easily recognisable to anyone working in National Health Service (NHS) mental health services in 2021 and whose origins demand scrutiny. Perhaps the most widespread example is the language of suicide risk.

Despite all evidence against checklists and risk stratification,^4^ we continue to behave as if risk is both predictable and quantifiable, persuading ourselves that certain stock phrases convey a protective coating. ‘Fleeting thoughts of suicide’, for example, sometimes seen as the precursor to an ‘impulsive’ suicide attempt or act of self-harm. Although it is not without value to record these things in the course of trying to understand someone's state of mind, it is important to question the attached meaning. In writing ‘no plans or intent’ we make ourselves feel better about the unpredictable nature of suicide, hanging false hope on thoughts that come and go. Rather than admit that someone might end their life but we don't know when or how, we purport to know it is unlikely to occur. This inevitably leads us to seek reassurance from patients, framing questions about suicide for a negative answer.^5^ Rarely is this more clear than when documenting that someone can (or cannot) ‘guarantee their safety’. It should not be for patients to ‘guarantee’ anything to clinicians – it is our responsibility to hold hope, not for them to promise us a future in which we are not standing before a coroner. It can be painfully obvious when a clinician has alighted on their ‘aha’ moment, the thing that (they believe) proves low risk. Any suggestion that a person has ambivalence towards suicide or actually wants to live (such as voluntarily seeking medical help) risks this interpretation. It is almost as if we take the worst-case scenario and work backwards from there, starting at ‘this person might kill themselves’, followed by ‘how can I prove it wasn't my fault?’ Our starting point should be the simple recognition of distress and a desire to help. It is that connection on a human level which so often makes the difference to people in crisis. Nobody ever says they were saved by a thorough risk assessment, and certainly not one treated as a stand-alone list of questions delivered without empathy. One wonders if there is another medical specialty in which there is such dogged devotion to a non-evidence-based practice. We seem to pay heed to neither scientific evidence nor personal testimony, available in abundance: ‘When the focus is purely on risk, professionals are often left with the frustration and anxiety of holding the risk, service users are left invalidated and abandoned’.^6^ Bad experiences reinforce learning; inquests and internal investigations can feel adversarial and sometimes ask the wrong questions. Clinicians may feel pressured to say (with the benefit of hindsight) that they would have done things differently; perhaps the only acceptable excuse for having ‘got it wrong’, so to speak, is because we thought the risk was not there. Miles argues that many such problems have their basis in shame, which means that doctors (and other health professionals) ‘become morally neutral, unquestioning automatons, at the mercy of organisational edicts, and fail to advocate for the needs of their patients’.^7^

Further speculation seeks not only to see the future but to determine a person's motivation. Someone who frequently harms themselves in a way that may or may not have suicidal intentions may be pronounced at risk of ‘death by misadventure’. The implication here is that the patient may die without really meaning to. It is impossible to forecast the drivers and intentions behind a theoretical final act; attempting to do so is designed to alleviate our anxiety about the opacity of the future and introduce a seed of blame on the part of the patient. Similarly, patients may find themselves told they ‘have capacity’ to end their lives, in a perverse justification of medical inaction.^8^ Mental capacity – a concept enshrined in legislation intended to enhance the autonomy and decision-making of vulnerable people – is used to legitimise neglect.^9^ The tendency to stray from objectivity into value judgement is an unfortunate but familiar feature of psychiatric notes, from the truly offensive ‘manipulative’ to the frankly bizarre ‘behavioural’, used to infer that a patient is doing something in a conscious and deliberate manner rather than because they can't help it. Such terminology does not stand up to scrutiny: not only does it arrogantly assume the ability to precisely determine patients’ motivations and intentions, but it is also nonsensical. All behaviour is ‘behavioural’; one might as well describe breathing as respiratory.

Moral judgement and focus on self-preservation are both ways in which we make the case for denying people care. We do a further massive disservice to patients by assuming they do not see through us, perpetuating the sense of alienation which characterises too many encounters with mental health services.^2,10^

## Systems designed to exclude

We cannot blame individuals for mindless practice without acknowledging the system that has created them. Ours is a culture of senseless fragmentation: separation of addiction and illness, of mental and physical, of mind and brain, of deserving and undeserving. At their very worst, modern mental health services seem to operate on two polarised (but related) values: coercion and exclusion. The former is probably most familiar as a critique of psychiatry as per the recent government White Paper.^10^ Time will tell whether statutory reform will lead to the desired aim of reduced coercion. Less talked about, perhaps, than the coercive aspect of psychiatry is the troubling drive to exclude people from services. We exclude based on postcode, diagnosis, complexity, comorbidity. Too much need, not enough need. Risk, lack of motivation, readiness for change, any possible reason to keep people out. We have apparently accepted, without question, the term ‘gatekeeping’ to refer to admission to psychiatric beds, a process that generally involves the agreement of a crisis resolution and home treatment team. While accepting the almost perpetual state of bed crisis in NHS psychiatric services, we should consider what this terminology says about the systemic attitude towards our patients. Our services are fortresses; patients are intruders to be prevented from breaching our defences. There is a rot in a system that views beds as needing protection from patients. Mental health services have developed an ethos of exclusion at an organisational level which naturally drives and perpetuates poor practice at an individual level.

One form of exclusion from care arises out of the false distinction between mind and body. Referrals to secondary care mental health services may be refused if there is a perception that the problem is ‘organic’ unless it fits neatly into a memory clinic remit. René Descartes died in 1650 yet still we subscribe to the dualistic fantasy that mental and physical can be clearly delineated, with health services persistently commissioned in a way that keeps them separated. This has resulted in baseless and ill-defined concepts which dominate practice: take ‘medical clearance’, for example – a requirement that any patient attending an emergency department be reliably pronounced to have no medical condition before having a psychiatric assessment.^11^ Although nobody would argue that an acute medical condition should not be promptly identified and treated, problems arise when we refuse to assess and manage in parallel. As is now recognised, this leads to inadequate patient care and should not be routine practice^12^ (though its grip is tenacious). We ask medical colleagues to ‘exclude organic causes’ of a disturbed mental state, as if it is always possible to do this acutely or to precisely isolate which symptom arises from which condition; as if conditions cannot coexist. In their detailed analysis of the incoherent distinction between ‘organic’ and ‘functional’, Bell et al^13^ conclude that ‘the functional–organic distinction often seems like a tool that helps determine treatment priority dressed up in the language of causation’. Neurologists and other specialists may be as guilty of this as psychiatrists,^14^ but we should surely have a greater interest in challenging what is essentially another form of stigma. As long as we rely on outdated pseudo-medical concepts, mental health workers will view ‘physical health’ as a kind of unpredictable bogeyman to be feared and avoided.

## A convenient pandemic

Stigma towards people with mental illness in medical settings is well-documented and tackling it a slow process; it was only in January 2020 that the Side by Side consensus statement was published^13^ but the COVID-19 pandemic that hit us just a month later jeopardises its intentions. Driven by the pandemic, there is a vogue for developing acute psychiatric assessment sites away from emergency departments.^15^ Across the UK are hastily created diversions for people in mental health crises and it remains to be seen whether these will prove either safe or cost-effective. While acknowledging a genuine need for infection control, the more cynical among us may see certain organisations leaping on the opportunity to do what they have been wanting to for years, which is to exclude psychiatric patients from emergency departments. The party line is that this is a compassionate move: an emergency department isn't the place for someone in mental health crisis.^16^ Arguably, it's not the most relaxing place for someone with sepsis or a fractured neck of femur either but it's where most of us would want to be in that situation. When Samuel Shem, in his cult novel about North American internal medicine,^17^ coined the term ‘GOMER’ (Get Out of My Emergency Room) he was describing a group of elderly patients with dementia, perceived to use time and resources but never improve or die. ‘GOMER’ refers to the doctors’ reaction when these unfortunate people arrive in their department. However, it seems as if this attitude is even more applicable to people with mental illness, certainly in the 21st-century NHS. Treating psychiatric patients as ‘GOMERs’ is systemically endorsed. A separation of mental and physical emergency locations reinforces the dangerous notion that we can reliably ascertain (even pre-hospital) whether someone needs ‘physical’ care or not.

The pandemic has also focused attention on the concept of moral injury – ‘perpetrating, failing to prevent, or bearing witness to acts that transgress deeply held moral beliefs and expectations’^18^ – in medicine.^19^ There is a psychological toll that comes with having to ration resources, transfer patients out of area owing to bed pressures, suspend vital services and see waiting lists grow longer. These have been headline news over the past year,^20^ yet all are challenges that have faced psychiatry for far longer. Perhaps we have already grown used to excusing exclusion and senseless divisions in order to avoid the reality that services have been systematically cut and we cannot give patients what they need.^21^ The mental health profession has had its compassion eroded by moral injury for longer than we can remember, rationing care for so long that we have come to believe that exclusion is clinically indicated. We claim to be encouraging personal responsibility and autonomy, preventing dependence, avoiding institutionalisation, reducing unnecessary referrals, all of which allows systemic failings to continue. Some of our processes seem almost designed to harm; the ways in which institutional factors have an impact on specific aspects of people's illnesses Kafkaesque. For example, people with eating disorders, among whom the belief that one is ‘not sick enough’ is common, are literally denied help until they are ‘sick enough’.^22^ People with personality disorder diagnoses who have experienced trauma, rejection and interpersonal discord throughout their lives are rejected by professionals within a system that tells them they should not have time and resources wasted on them.^23^ These are not cognitive distortions but grim reality. This culture of exclusion, coupled with the expectation that patients take responsibility to quell clinician anxiety, is a toxic mix.

## Rehumanising psychiatry

One consultant psychiatrist pontificating about culture in a journal is not going to drive the kind of genuine change that needs to filter through every layer of our system. Decades of damage requires time to repair, not to mention the buy-in of all parties. Training has a role, from undergraduate level upwards across all professional groups, but organisations must have the guts to implement culture shift rather than a series of slightly altered tick boxes. The more clinicians work side by side with the people who use mental health services (and those who have been excluded from them), the more effective the message. Meaningful training and service development should be truly co-produced; a fundamental problem is the focus on beds and breaches and targets instead of the human story behind each number. Arguably, senior managers who would willingly allow an unwell patient to wait in an emergency department for more than 24 h for the purposes of ‘gatekeeping’ might take a different view if it were them or a loved one, so should not be permitted to distance themselves from clinical realities. This is not to say that all those working on the front line are faultless patient advocates. Lack of compassionate care for people in mental health crisis pervades emergency services and, although burnout and ‘compassion-fatigue’ play a role, there are deep-seated negative attitudes towards certain patients. Although co-production is vital, the responsibility for recognising and calling out harmful culture and practices should not fall entirely on the shoulders of those who have suffered it. In short, our profession must open its eyes. Regulatory bodies such as the General Medical Council tell us to ‘make the care of the patient your first concern’^24^ yet we stray from this to prioritise the needs of clinicians and organisations. In a culture of self-protection, exclusion will inevitably become a central aim because, of course, the best way to prevent ourselves from harm is to prevent the ‘danger’ from getting near us. How have we so comprehensively forgotten to put patients first?

Realism and honesty should be embedded in training, rather than teaching perfect medicine in an imperfect world. Our patients will have more trust in us if we are open about scarcity of resources and restrictions on referrals; if we acknowledge that we cannot provide all we would like to. Instead of pretending that exclusion is clinically appropriate we must name it. However, clinical staff can only safely preach honesty if senior leaders support this endeavour. Although the Royal College of Psychiatrists has produced some welcome position statements and guidelines, this must translate to institutional and organisational change. Senior consultants, managers and academics who do not recognise anything in this article may need to reacquaint themselves with the front line; it will surely resonate with junior doctors, nurses, allied health professionals, students and – most importantly – patients and carers: ‘Educating the next generations of clinicians and social workers is vital, but they won't survive immersion in toxic cultures. We need honesty from organisations where poor care and neglect have become systemic and endemic’.^25^

Patients and carers have been speaking out about exclusion and iatrogenic harm for too long; psychiatrists complaining about blame culture similarly. It is time this was translated into action by those with most power to effect change. Consider this a call to arms: if the content resonates then ensure you do more than shout into your echo chamber.

## Data Availability

Data availability is not applicable to this article as no new data were created or analysed in its writing.

## About the author

**Chloe Beale**, MBBS, MRCPsych, LLM, is a consultant liaison psychiatrist at Homerton University Hospital, London, trust lead for suicide prevention for East London NHS Foundation Trust and an honorary clinical senior lecturer at Queen Mary, University of London, UK.
